# Supplementary material for: Aspartate β-Hydroxylase Is Upregulated in Head and Neck Squamous Cell Carcinoma and Regulates Invasiveness in Cancer Cell Models
Source: Int J Mol Sci. 2024 May 3;25(9):4998. doi: 10.3390/ijms25094998 (PMC11084744; doi:10.3390/ijms25094998)
Supplement: Supplementary file 1 [file ijms-25-04998-s001.zip › ijms-2983362-supplementary.pdf]

Supplementary

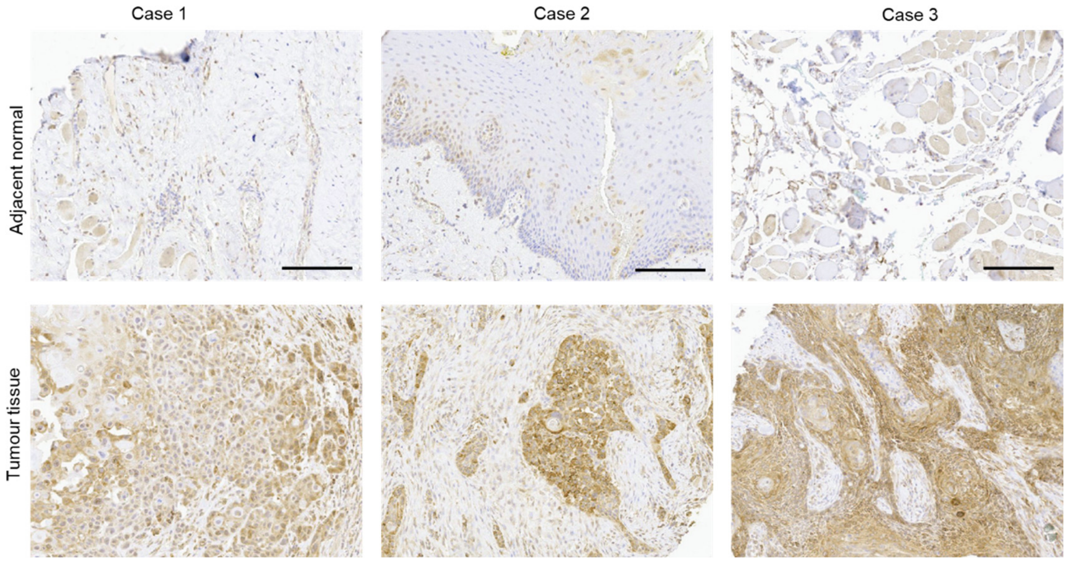

Figure S1: Representative images of magnified sections from ASPH staining of HNSCC tumor tissues along with adjacent normal tissues. Clear indications of higher ASPH expression is seen in tumor tissues with a uniform distribution in epithelial and stromal sections.

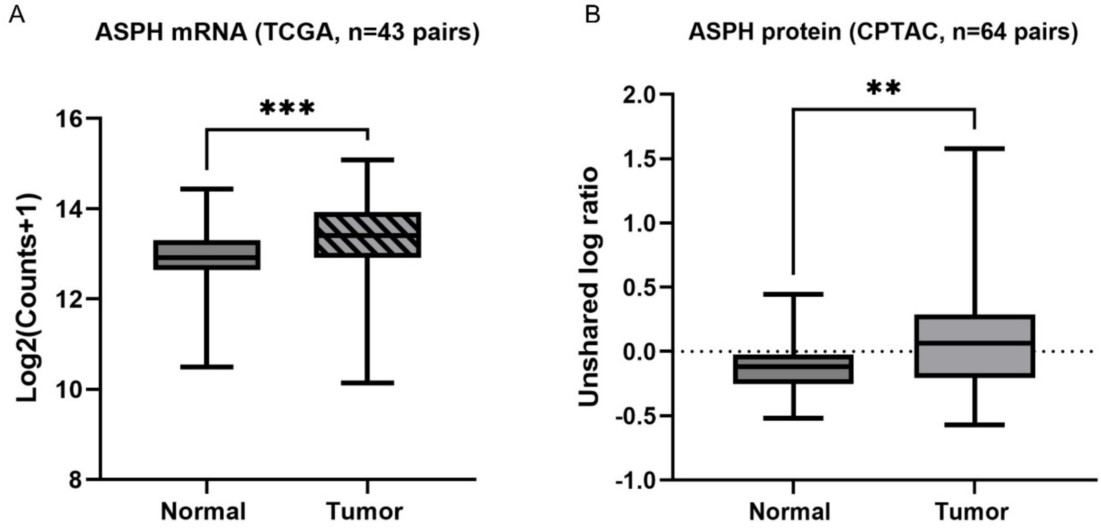

Figure S2: ASPH is elevated in tumors of HNSCC patients. (A) ASPH mRNA in tumor compared with paired normal tissue (n=43) of HNSCC patients in the TCGA dataset. (B) ASPH protein in the tumors and adjacent normal tissues of HNSCC patients (n=64) in the CPTAC dataset. Statistical significance using paired t-test; **P value<0.001, ***P value<0.0001

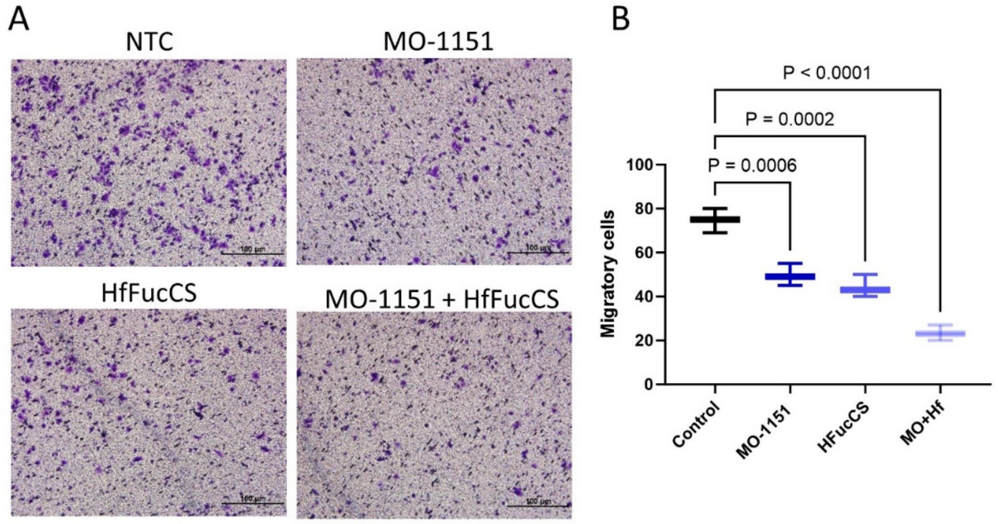

Figure S3: (A) Representative images of inserts from Transwell migration chamber after 24 hrs of cell migration. NTC shows highest migration of the control SCC35 cells. Treatments on inserts with MO-I-1151, HfFucCS and their combination were carried out as indicated. Inserts were stained with crystal violet cell stain before imaging. Scale bars are 100µm. (B) Quantification of migratory cells in each condition. Treatment with MO-I-1151 showed significant reduction in cell number (33%, P=0.0006) compared to control which was further reduced by about 65% upon combination treatment with HfFucCS.
